# Supplementary material for: SARS-CoV-2 seroprevalence and risk factors among meat packing, produce processing, and farm workers
Source: PLOS Glob Public Health. 2022 Jul 13;2(7):e0000619. doi: 10.1371/journal.pgph.0000619 (PMC10022315; doi:10.1371/journal.pgph.0000619)
Supplement: S1 Table — (DOCX) [file pgph.0000619.s001.docx]

**S1 Table. ELISA Validation Data**

|  | **% Sensitivity (95% CI)** | **% Specificity (95% CI)** |
| --- | --- | --- |
| RBD total Ig ELISA (1:40)  n=69 positive controls  n=122 negative controls | 85.5% (75.3, 91.9) | 99.2% (95.5, 100.0) |
| RBD total Ig ELISA (1:120)  n=16 positive controls  n=20 negative controls | 100% (80.6, 100) | 100% (83.9, 100.0) |

RBD: receptor-binding domain; ELISA: enzyme-linked immunoassay; CI: confidence interval;
