# Supplementary material for: SARS-CoV-2 seroprevalence and risk factors among meat packing, produce processing, and farm workers
Source: PLOS Glob Public Health. 2022 Jul 13;2(7):e0000619. doi: 10.1371/journal.pgph.0000619 (PMC10022315; doi:10.1371/journal.pgph.0000619)
Supplement: S2 Table — (DOCX) [file pgph.0000619.s002.docx]

**S2 Table. Food processing occupational characteristics associated with SARS-CoV-2 seropositivity stratified by industry (fall 2020)**

|  | Meat packing | |  | Farming | |  |
| --- | --- | --- | --- | --- | --- | --- |
|  | Seropositive (n=31) | Seronegative  (n=17) | RR (95% CI) or p-value | Seropositive  (n=27) | Seronegative (n=33) | RR (95% CI) or p-value |
|  | No. (%) | |  | No. (%) | |  |
| Mask wearing | 27 (87%) | 16 (94%) | 0.78 (0.48-1.29) | 20 (74%) | 20 (61%) | 1.43 (0.73-2.80) |
| Eye protection | 14 (45%) | 6 (35%) | 1.15 (0.76-1.74) | 6 (22.2%) | 3 (9%) | 1.62 (0.92-2.85) |
| Protective clothing | 27 (87%) | 15 (88%) | 0.96 (0.52-1.77) | 7 (25.9%) | 10 (30.3%) | 0.86 (0.46-1.70) |
| Shoe covers | 11 (35%) | 0 (0%) | **0.005** | 1 (3.7%) | 1 (3.0%) | 1.14 (0.28-4.70) |
| Shield | 22 (71%) | 9 (53%) | 1.34 (0.81-2.21) | 6 (22.2%) | 2 (6.1%) | **1.82 (1.09-3.06)** |
| Frequent hand washing | 28 (90%) | 17 (100%) | 0.19 | 17 (63.0%) | 18 (54.5%) | 1.21 (0.67-2.19) |
| Indoors | 27 (87%) | 15 (88%) | 0.96 (0.52-1.77) | 10 (37.0%) | 9 (27.3%) | 1.27 (0.72-2.22) |
| Crowded | 18 (58%) | 12 (71%) | 0.83 (0.55-1.25) | 6 (22.2%) | 3 (9%) | 1.62 (0.92-2.85) |
| Cold temperature | 24 (77%) | 13 (76%) | 1.02 (0.61-1.69) | 2 (7.4%) | 0 (0%) | 0.11 |
| High noise level | 25 (81%) | 11 (65%) | 1.14 (0.89-1.45) | 11 (40.7%) | 5 (15.2%) | **1.23 (1.02-1.49)** |

CI: confidence interval; RR: risk ratio; SD: standard deviation. Other food processing: includes farming and produce processing industries. Mask wearing: regularly wearing a mask at work. Eye protection: regularly wearing protective glasses or face shields at work. Protective clothing: regularly wearing protective clothing (such as a gown or apron) at work. Shoe covers: regularly wearing shoe covers at work. Shield: having a protective shield between the individual and other workers. Frequent hand washing: washing or sanitizing hands multiple times per day at work. Indoors: always working indoors. Cold temperatures: below 16°C. Crowded: being close enough to touch another worker without walking. High noise level: loud enough to necessitate yelling to communicate.
