# Supplementary material for: SARS-CoV-2 seroprevalence and risk factors among meat packing, produce processing, and farm workers
Source: PLOS Glob Public Health. 2022 Jul 13;2(7):e0000619. doi: 10.1371/journal.pgph.0000619 (PMC10022315; doi:10.1371/journal.pgph.0000619)
Supplement: Attachment — Submitted filename: Response to Reviewers.docx [file pgph.0000619.s003.docx]

**Manuscript ID** PGPH-D-21-01092

Title: “SARS-CoV-2 Seroprevalence and Risk Factors Among Meat Packing, Produce Processing, and Farm Workers”

**Response to reviewer comments**

Reviewer: 1

Comments to the Author

*“*I wish to commend the authors on their well written manuscript that was a delight to read. The authors have completed a noteworthy analysis of the risk factors of SARS-CoV-2 infection among a key occupational group in an insightful and considerate way. Small mentions of this appear in the methods that demonstrate the lengths the authorship team went to gather data. First starting with enrollment at a federally qualified health center, then engaging local community organizations and traveling to participants residences to for study visits. The authors also followed their participants weekly and referred them to local clinics for additional testing. This study demonstrates a careful and considerate analysis of a vulnerable population while also significantly contributing to the literature.
I offer a few suggestions that I think will make the manuscript stronger*.”*

- We thank the reviewer for his or her comment. This study was a meaningful endeavor to us, and it was a privilege to work together with our local community during this challenging time.

Major concerns

1. Major concern: The authors mention repeat biological sampling from their study participants and it is not clear what samples were used in the analysis or the date/range for the estimates of seroprevalence. For example, did the authors consider all samples when evaluating seroprevalence of SARS-CoV-2 or just the first one w/ enrollment. It is not clear in the manuscript as written, what time window their results are from and as a result, the conclusions will have limited impact in this extended pandemic with multiple variants and shifting measurement/containment practices if it is not clear what the exact time period or range the authors have measured.
   a. Example: Statistical analysis: “The primary outcome of interest was SARS-CoV-2 positivity” by what time point?

- Thank you for letting us know that the timeline was not clear. All participants performed an enrollment visit between September and December of 2020. Participants were also invited to complete monthly follow-up visits, where repeat specimens were collected. In this manuscript, we only report data from enrollment visits. To clarify this, we made the following changes in the text:

[Methods] Participants completed an enrollment visit between September and December 2020 consisting of a questionnaire to collect data on demographic characteristics, medical history, workplace and household characteristics, and preventive behaviors; three biological samples (blood, saliva, and nasal turbinate swab) were collected as well.

We deleted the following sentence: Participants were invited to complete monthly follow-up visits, where the same specimens were collected, and a short follow-up questionnaire was administered to update exposure and clinical data.

[Discussion] The prevalence of SARS-CoV-2 antibodies in our study population was 50.0%, over five times the rate observed in most of the United States when we conducted this study in fall 2020.

b. The authors mention in the first time of discussion “when we conducted this study in September 2020- but in another section of the paper refer to September – December for enrollment of participants.

Please see comment above.

c. It appears as if the data analyzed do not include the monthly follow-up visits. Should these results be included in the methods if they are not used?

Please see comment above.

d. Enrollment criteria – working since “February 2020” I imagine this is just the cut off for being employed prior to the start of the pandemic but it might be good to explicitly state this.

- Yes, that was the intention. We made the following clarification: “Adults (age ≥18 years) who had resided and worked for at least two weeks in a meat packing plant, produce processing facility, or commercial farm in North Carolina since February of 2020 (when the first nontravel-related COVID-19 cases were identified in the US) were eligible to participate in the study.”

2. I think the authors have placed a bit too much emphasis on statistical significance given their small sample size. “When cold work environment was added to the adjusted analysis (Model 2), the association of SARS-CoV-2 seropositivity with high noise levels and cold environment lost statistical significance.” While this is true the magnitude was still notable and the lower CI was 0.94. As written, with no additional interpretation- it dismisses the association as if it were null. It is good to caution against over interpreting- but the authors see attenuation of their association which is expected.

- We agree that our relatively small sample size should caution against over-interpretation of loss of statistical significance in this analysis. To clarify this, we left the analyses in Table 2 unchanged, but removed the sentence referenced above.

3. Tables need more descriptive titles such that they can stand alone from the paper. Please consider adding dates, locations and other descriptive measures of the study population to the table. Also please see comment 1 about when exactly seroprevalence was measured- this should be clear across the manuscript text and in the table(s) and supplement.

- We made the following updates to the table titles:

**Table 1. North Carolina food processing worker characteristics by industry (fall 2020)**

**Table 2. North Carolina food processing worker and workplace characteristics associated with SARS-CoV-2 seropositivity (fall 2020)**

4. Authors mention a supplement but there was no supplement file available in the peer review system. As such- I was unable to review the supplemental results.

- Our supplement has been added.

Minor thoughts

1. When farm workers were most likely to have SARS-CoV-2 antibodies without a history of COVID-19 illness- is there any literature or anecdotal knowledge about the inability to distinguish low/moderate symptoms of COVID-19 illness with other farm laborer sickness (i.e. pesticide exposure, heat stress) or work-related fatigue? I wonder if that may also be a reason why fewer illnesses were reported in this group.

- We are not familiar with any literature addressing this, but we agree this could contribute to higher rates of SARS-CoV-2 seropositivity without known infection. We added the following statement: Farm workers also frequently experience non-specific respiratory symptoms and fatigue from occupational exposures that could be difficult to distinguish from COVID-19 illness [24].

2. Also relevant to discussion- are there any studies/anecdotal knowledge about cold environments and nasal discharge (runny noses) that also may increase the spread of COVID-19?

- We are not familiar with any literature addressing this topic.

3. Page 4- liner 101-102 “Which has one of the food processing industries in the US” – seems like a word or two is missing from this phrase. Please revise for clarity.

- We thank the reviewer for noticing this error. We made the following revision: “Therefore, we conducted an observational cohort study of meat packing plant, farming, and produce processing workers in North Carolina, which has one of the largest food processing industries in the US.”

Reviewer: 2

Comments to the Author

“This manuscript furthers understanding of COVID-19 transmission in food processors, a large yet understudied group. Of particular interest and importance is the linkage between noise levels and transmission. See the attached documents for comments and minor revisions.”

- We thank the reviewer for his or her comments.

1. Can you mention the demographics of those who were excluded from your study? Are there any demographic/social differences between those excluded and those who were included in the observational group? A brief discussion of potential selection bias or how you avoided such would be a great addition to the methods.

- Unfortunately, we did not have a protocol to formally analyze the demographics of excluded individuals, in part because we could not ask for personal details from individuals prior to being consented. In my personal experience as study coordinator, I received many phone calls from interested individuals who had seen our flyers but did not qualify because they did not work in food processing.

2. Is concentration of antibodies important? This study only looks at presence/absence of antibodies. Please discuss this choice of methods (looking at presence/absence only) and what could be gained/lost by looking at concentration differences across study participants.

- As our study was cross-sectional, we were unable to determine the timing of participants’ infection, making antibody concentration difficult to interpret. Therefore, we focused on the presence or absence of antibodies to consider the role of sociodemographic and behavioral variables in infection risk.

3. What was the lower limit of detection for seropositivity and is it possible that some of the study population fell below the limit of detection at the time of sampling? How would this have impacted results?

- Samples for this study were collected through December 2020. As the first case of COVID-19 was identified in North Carolina on March 3, 2020, participants were presumably infected within ten months of testing. Meat packing plants in North Carolina experienced significant outbreaks in April and May 2020, so most participants were likely infected within six to nine months of testing. Prior studies have demonstrated persistence of detectable SARS-CoV-2 antibodies for six to nine months after infection [1, 2]. We added the following sentence to our discussion of limitations: It is also possible that our ELISA missed a few early cases due to antibody waning. We estimate this represents a very small number of our participants, as all participants were tested within 10 months of the first identified COVID-19 case in North Carolina, and prior studies have demonstrated durable antibody response for at least six to nine months [34,35].

34. Haveri A, Ekström N, Solastie A, et al. Persistence of neutralizing antibodies a year after SARS-CoV-2 infection in humans. Eur J Immunol 2021; 51(12):3202-3213

35. Lumley, Wei, O’Donnell, et al. The duration, dynamics, and determinants of severe acute respiratory syndrome coronavirus 2 (SARS-CoV-2) antibody responses in individual healthcare workers. Clin Infect Dis 2021; 73(3):e699-e709.

1. What non-occupational related activities/behaviors for specific worker groups could have influenced transmission and how were these factors controlled for?

- Our study also considered several non-occupational characteristics, including age, sex, ethnicity, smoking status, and frequency of in-person socialization. These factors were included in our analysis but were not found to be significantly associated with SARS-CoV-2 seropositivity (Table 2).

2. High noise levels are defined as “being loud enough to necessitate yelling”. How was this measured? Were the workers interviewed on whether they needed to yell to communicate during shifts? Was yelling observed?

- As we were unable to visit any of the workplaces to quantitatively measure noise levels (despite multiple requests to local meatpacking plants), our analysis relied on self-reported data from participants. The enrollment questionnaire asked participants about occupational noise levels, and the description “loud enough to necessitate yelling” was used to quality noise levels in a simple, understandable way.
